# Multi-locus genome-wide association analysis of grain yield, plant height, panicle length, and flowering time in the Senegalese pearl millet [*Pennisetum glaucum* (L.) R. Br.] germplasm

**DOI:** 10.1371/journal.pone.0358045

**Published:** 2026-10-06

**Authors:** Aliou Ba, Oumar Diack, Thomas L. Odong, Safiétou Tooli Fall, Richard Edema, Arfang Badji, Adama Faye, Tonny Obua, Mildred Ochwo-Ssemakula, Paul Gibson, Mamadou Mar, Jessica B. Bacourine, Yagouba Diao, Mahoussi K. Arnaud Djanta, Ndjido A. Kane, Phinehas Tukamuhabwa

**Affiliations:** 1 Makerere University Regional Centre for Crop Improvement (MaRCCI), College of Agricultural and Environmental Sciences, Makerere University, Kampala, Uganda; 2 Department of Crop Science and Horticulture, College of Agricultural and Environmental Sciences, Makerere University, Kampala, Uganda; 3 Centre National de Recherches Agronomiques de Bambey, Institut Sénégalais de Recherches Agricoles, Bambey, Senegal; 4 Department of Soil Science and Land Use Management, College of Agricultural and Environmental Sciences, Makerere University, Kampala, Uganda; 5 Centre d’Étude Régional pour l’Amélioration de l’Adaptation à la Sécheresse, Institut Sénégalais de Recherches Agricoles, Thiès, Senegal; University of Florida Tropical Research and Education Center, UNITED STATES OF AMERICA

## Abstract

Pearl millet [*Pennisetum glaucum* (L.) R. Br.] is a staple cereal underpinning food and nutritional security across the semi-arid Sahel, yet locally calibrated genomic resources for its improvement in West Africa remain scarce. Improving its key agronomic traits is therefore a breeding priority, despite the crop’s inherent hardiness. This study characterized the genetic architecture of four agronomically decisive traits: grain yield, plant height, panicle length, and flowering time, in a Senegalese panel combining internationally derived PMiGAP inbred lines with locally adapted breeding material. Genotypes were evaluated across multiple Senegalese locations between July and October 2024 and genotyped on the DArTseq platform, yielding 14,718 high-quality SNPs. Analysis of variance revealed highly significant genotypic effects, with broad-sense heritability ranging from 0.75 to 0.91. Plant height, panicle length, and grain yield were positively correlated, whereas flowering time was negatively correlated with yield, consistent with an early-flowering drought-escape strategy. The association panel showed weak population structure (k = 2), and linkage disequilibrium decayed rapidly, falling below r^2^ = 0.1 at approximately 8 kb. Genome-wide associations were tested in a subset of 137 stable genotypes using a multi-locus mixed-model approach with correction for population structure and kinship. Eleven marker–trait associations were detected across six of the seven pearl millet chromosomes (LOD 6.83–10.78), explaining 5.92% to 48.67% of phenotypic variance, with plant height the most extensively resolved trait. Candidate-gene annotation linked several markers to genes plausibly involved in cell expansion, inflorescence development, and hormone-mediated growth, including an expansin, an AP2/ERF transcription factor, and an auxin-conjugating glucosyltransferase. Flowering time associated with a single chromosome-2 locus implicated in auxin homeostasis, on the same chromosome as PHYTOCHROME C (PHYC), a principal flowering-time determinant in the crop. Together, these associations provide candidate targets for marker-assisted and genomic selection and offer a foundation for genomics-assisted pearl millet improvement in Senegal and the broader West African Sahel.

## Introduction

Pearl millet [*Pennisetum glaucum* (L.) R. Br.] is a C4 cereal widely cultivated across the Sahelian belt of West Africa and the Indian subcontinent, where it sustains rain-fed crop-livestock farming systems under conditions of high temperature, erratic rainfall, and low soil fertility [1]. Originated from tropical Western Africa between 4,000–5,000 years ago, the crop differentiated into two major races: the *globosum* race, which spread westward, and the *typhoides* race, which reached East Africa before extending to India and southern Africa some 2,000–3,000 years ago [2]. Beyond its adaptation to harsh environments, pearl millet is nutritionally outstanding, with high contents of carbohydrates, protein, fat, dietary fibre, iron, and zinc, and a low glycemic index of approximately 50, positioning it as an important candidate for functional-food markets worldwide [3,4]. It ranks sixth among the world’s major cereals [5] and plays a central role in food security and farmer livelihoods across Sub-Saharan Africa, where it is consumed in diverse forms including porridges, couscous, and breads [6]. In Senegal, pearl millet is cultivated on a substantial portion of the national cereal area, with an average productivity of approximately 1,353 kg/ha, higher than the West African average of 719 kg/ha but still far below the potential yield of 3,000 kg/ha achievable under research conditions [7]. This yield gap is driven by a combination of poor soil conditions, limited improved seed availability, erratic rainfall, and increasing temperatures [7,8]. Farmer preference studies across West Africa consistently identify grain yield and its components, early flowering, downy mildew resistance, and drought tolerance as the top priorities for varietal improvement [9,10], highlighting the need for breeding strategies that simultaneously address productivity and adaptation to variable Sahelian environments. The Senegalese national pearl millet breeding program, coordinated by the Institut Sénégalais de Recherches Agricoles (ISRA), has developed a range of advanced generation populations targeting these farmer-preferred traits. These include ISRA MIL inbred lines (ISMI lines), derived from improved early-flowering Senegalese cultivars and introduced germplasm; ISRA Millet Population lines (ISMP), developed from Senegalese and West African elite parents; and Pearl Millet Landraces of Senegal lines (PLS), derived from late-flowering Senegalese landraces. In addition, the program conserves the Pearl Millet Inbred Germplasm Association Panel (PMiGAP), a reference diversity panel comprising accessions from over 20 countries maintained at the International Crops Research Institute for the Semi-Arid Tropics (ICRISAT), which captures a wide range of genetic diversity for agronomically important traits [11,12]. Together, these materials constitute a strategic germplasm base for dissecting trait architecture and identifying novel alleles for crop improvement. However, genomic research on these Senegalese populations has remained limited, with few studies exploring genome-wide genetic diversity, and opportunities to accelerate genetic gains through genomic-assisted breeding have therefore been largely untapped [9,13]. Genome-wide association studies (GWAS) have emerged as a powerful tool for dissecting the genetic architecture of complex agronomic traits, offering higher resolution, broader genome coverage, and the ability to exploit historical recombination events compared to biparental QTL mapping approaches [14,15]. While conventional single-locus GWAS methods are prone to false positives in structured populations, multi-locus GWAS models reduce this limitation by simultaneously fitting multiple markers, enabling more reliable identification of small- to moderate-effect loci for polygenic traits [16]. GWAS has been successfully applied to pearl millet for nutritional biofortification, yield-related traits, and stress tolerance [11,17], and recent advances in pan-genomics and multi-omics resources are further expanding the functional genomics toolkit available for this species [18,19]. Beyond marker discovery, the functional interpretation of GWAS loci increasingly benefits from integration with complementary layers of omics evidence, including transcriptomic, epigenomic, chromatin-accessibility, and cis-regulatory data, that can connect associated intervals to the genes and regulatory elements driving trait variation. This is now becoming tractable in pearl millet where single-cell transcriptomics has resolved cell-type-specific, stress-responsive expression patterns in the crop [20]. Furthermore, the scPlantReg framework and database integrate plant single-cell chromatin-accessibility profiles with cis-regulatory information, including cell-type-specific accessible chromatin regions and regulatory elements associated with transcription-factor families such as AP2/EREBP [21]. Complementary resources such as the Plant Chromatin Accessibility Database (PlantCADB) provide broader cross-species information on accessible chromatin regions, transcription-factor binding motifs, regulatory elements, associated genes, and SNPs. Together, these resources offer a direct route to integrate GWAS signals with cell-type-specific regulatory landscapes, prioritize candidate genes and regulatory elements, and guide their functional validation, including the transcription-factor candidates identified in the present study. Despite these advances, GWAS studies anchored to Senegalese germplasm and targeting the specific trait priorities of West African breeding programs remain rare, limiting the regional applicability of globally generated marker-trait associations. To address this gap, the present study characterized the genetic diversity and population structure of a collection of genotyped pearl millet accessions from the Senegalese germplasm, spanning locally developed breeding lines and internationally sourced PMiGAP material. To ensure that genotypic values could be reliably estimated and replicated across environments, marker–trait association (MTA) analysis was restricted to a panel of genetically advanced and stable entries. The specific objectives of this study were therefore to: (i) assess the genetic diversity of the overall genotyped collection and characterize population structure within the GWAS panel only; (ii) identify marker–trait associations for grain yield, panicle length, flowering time, and plant height within the same panel; and (iii) annotate candidate genes underlying the detected associations to inform functional validation and molecular breeding priorities in the Sahelian production context.

## Materials and methods

### Plant material

Three pearl millet germplasm sets maintained in the Senegalese national collection (ISRA) were used, each evaluated in a separate trial; detailed pedigrees are provided in **Supporting Information**. The first set was a diversity panel of 290 genotypes from multiple breeding sources: 63 PMiGAP inbred lines, a reference panel from ICRISAT [12], 158 F5 ISMI (ISRA MIL) lines from improved early-flowering Senegalese cultivars and introduced germplasm, 10 F3 ISMP (ISRA Millet Population) lines from Senegalese and West African improved parents, 29 F4 PLS (Pearl Millet Landraces of Senegal) lines from late-flowering landraces, and 30 checks and other accessions. Checks represented contrasting phenotypic extremes: Gawane and Thialack 2 (positive/negative for flowering time), SL423 and Souna 3 (grain yield), and SL28 and IBV8004 (panicle length). The second and third sets were segregating populations advanced by pedigree selection with recurrent selfing, comprising 90 F1 genotypes (F1-Population) and 50 F2 genotypes (F2-Population), respectively, each derived from several parental crosses.

### Field experimental design and phenotypic evaluation

Field work was conducted on experimental plots belonging to the Institut Sénégalais de Recherches Agricoles (ISRA) at the Centre National de Recherche Agronomique (CNRA), Bambey, Senegal. No field permits were required for this study, as all data collection was carried out on institutional research fields managed by ISRA, and access was granted by the institution. Trials were conducted between July and October 2024 across four locations in Senegal: Nioro du Rip (13°45′N, 15°48′W), Bambey (14.69°N, 16.45°W), Sinthiou Malem (13.77°N, 13.67°W), and Sefa (12.45°N, 15.32°W). The diversity panel was evaluated across all four locations using an alpha-lattice design (29 × 10) with three replications and a total surface area of 3302.85 m^2^ per location. F_1_-Population was tested at two locations, Nioro du Rip and Bambey (but only Bambey data are herein analyzed for reliability purposes), using an alpha-lattice design (9 × 10) with three replications and a total surface area of 931.5 m^2^ per location. F_2_-Population was evaluated across all four locations using a randomized complete block design (RCBD) with three replications and a total surface area of 2146.08 m^2^ per location. Land preparation and agronomic management followed standard protocols across all sites to ensure uniform crop establishment and minimize environmental variability. Prior to sowing, fields were cleared, plowed, harrowed, and leveled to obtain a fine and uniform seedbed. Plots were laid out according to the respective experimental designs, and sowing was performed at a recommended spacing of 40 cm between hills and 75 cm between rows. Nitrogen Phosphorus Potassium (NPK-15-15-15) fertilizer was applied in two split doses, at planting and again 15 days after planting at a dose of 50 Kg/ha. Weed, pest, and disease management were carried out uniformly across all plots in accordance with local agronomic recommendations.

### Data collection

Although multiple agronomic traits were recorded during the trials, the present study focused on four key traits: grain yield, panicle length, and plant height and flowering time. Data were collected at appropriate growth stages following standard practices to ensure accuracy and consistency across all sites. Flowering time was recorded as the number of days from sowing to 50% anthesis at the plot level, defined as the date on which at least half of the plants in a plot had begun shedding pollen from the primary spike. Grain yield was assessed at physiological maturity, once the crop had reached full grain fill and moisture content had stabilized; panicles were harvested plot-by-plot, threshed, and cleaned before weighing, with grain weight converted to kilograms per hectare using the effective harvested plot area and adjusted to a standard moisture content of 12.5% to allow valid comparisons across sites and harvest dates. Panicle length and plant height were both measured at physiological maturity on a subsample of three randomly selected plants per plot, with the mean value used as the plot-level observation. Panicle length was measured from the base of the primary panicle, immediately above the flag leaf node, to the tip of the terminal spikelet using a graduated ruler, with measurements restricted to primary panicles to avoid confounding effects of tillering. Plant height was recorded from ground level to the tip of the primary panicle, including the panicle.

### Statistical analysis of phenotypic performance

A linear mixed model with no fixed effects beyond the intercept was fitted to estimate variance components, with all factors included as random effects, according to the following equation:


$$\begin{array}{c} y_{ijkl}=\mu +(1|G_{i})+(1|L_{j})+(1|G\times L{)}_{ij}+(1|R_{k(j)})+(1|B_{l(kj)})+\varepsilon_{ijkl} \end{array}$$ (1)

Where:

- **y**_**ijkl**_ is the observed phenotypic value,
- **μ** is the overall mean,
- **(1|G**_**i**_) is the random effect of the i^th^ genotype,
- **(1|Lj)** is the random effect of the j^th^ location,
- **(1|G × L) ij** is the random effect of the genotype-by-location interaction,
- **(1|Rk(j))** is the random effect of the k^th^ replication nested within the j^th^ location,
- **(1|B l(kj))** is the random effect of the l^th^ block nested within the k^th^ replication and j^th^ location
- **ε**_**ijkl**_ is the residual error term, assumed to follow a normal distribution with mean zero and constant variance.

This full model was applied to the diversity panel, which was evaluated across the four locations, allowing estimation of all variance components including the genotype-by-location interaction. Because the F1-Population was evaluated at a single site and the F2-Population comprised segregating, non-fixed entries, variance components and broad-sense heritability were not estimated for these generations; they were summarized descriptively instead (see below). Broad-sense heritability for the diversity panel was calculated from the variance components using the following formula:


$$\begin{array}{c} H^{2}=\frac{\sigma_{G}^{2}}{\sigma_{G}^{2}+\frac{\sigma_{G\times L}^{2}}{n_{L}}+\frac{\sigma_{e}^{2}}{n_{L}n_{R}}} \end{array}$$ (2)

Where:

- $\sigma_{G}^{2}$= genetic variance (for genotypes)
- $\sigma_{G\times L}^{2}$ = genotype × location variance (where applicable)
- $\sigma_{e}^{2}$ = residual variance
- $n_{L}$ = number of locations
- $n_{R}$= number of replications per location

For GWAS, the Best linear Unbiased Estimators (BLUEs) for all the genotypes were used as phenotypic data using a linear mixed model where genotype was treated as fixed and location, block and replication as random. The model was written as follows:


$$\begin{array}{c} y_{ijkl}=\mu +G_{i}+(1|L_{j})+(1|G\times L{)}_{ij}+(1|R_{k(j)})+(1|B_{l(kj)})+\varepsilon_{ijkl} \end{array}$$ (3)

Where:

- **y**_**ijkl**_ is the observed phenotypic value,
- **μ** is the overall mean,
- **G**_**i**_ is the fixed effect of the i^th^ genotype,
- **(1|Lj)** is the random effect of the j^th^ location,
- **(1|G × L) ij** is the random effect of the genotype-by-location interaction,
- **(1|Rk(j))** is the random effect of the k^th^ replication nested within the j^th^ location,
- **(1|B l(kj))** is the random effect of the l^th^ block nested within the k^th^ replication and j^th^ location
- **ε**_**ijkl**_ is the residual error term, assumed to follow a normal distribution with mean zero and constant variance.

The coefficient of variation among genotypes was calculated as the standard deviation of genotype BLUEs divided by their mean, and was used to assess phenotypic variability across the panel. This statistic was used to assess the relative magnitude of phenotypic variability among genotypes in the GWAS panel independently of trait scale, and to flag traits with high residual variability that may affect association power. Pearson’s correlation was also computed between the four studied traits in the GWAS panel. All statistical analyses mentioned above were conducted using the *R software version 4.5.1* primarily employing the ***lme4*** package for mixed model analysis.

### DNA sample collection

Leaf tissue for genotyping was sampled from 279 individuals, combining 178 entries drawn from the 290-genotype diversity panel combined with 53 F1 and 48 F2 individuals from the two segregating populations. Genetic-diversity metrics were therefore computed on the overall 279-genotyped collection, while genome-wide association analysis was restricted to the 137 most advanced, genetically stable entries (PMiGAP inbred lines, F5 ISMI lines, and checks) selected from the genotyped material. Samples were collected using leaf punchers and placed directly into 96-well collection plates. To prevent cross-contamination between genotypes, punchers were systematically sterilized with 70% ethanol prior to each successive sampling. Collected samples were then shipped to SEQART AFRICA, hosted at the International Livestock Research Institute (ILRI) in Nairobi, Kenya, for genotyping. DNA extraction was performed from leaf tissue using the *NucleoMag Plant DNA Extraction Kit* following the manufacturer’s protocol. The quantity and quality of extracted genomic DNA were assessed by gel electrophoresis on a 0.8% agarose gel, and DNA concentrations were confirmed to fall within the range of 50–100 ng/µl, meeting the quality thresholds required for downstream genotyping applications.

### Genotyping method

Genotyping was performed using the DArTseq Genotyping-by-Sequencing (GBS) platform, which provides rapid and high-quality genome-wide profiling. Library preparation followed the protocol described by Kilian et al. (2012) [22]. Briefly, complexity reduction was achieved through double digestion of genomic DNA using a combination of *PstI* and *HpaII* restriction enzymes, followed by ligation of barcoded adapters and a common adapter. Adapter-ligated fragments were then amplified by PCR to generate the final sequencing libraries. Sequencing was carried out on an Illumina NovaSeq X instrument using single-read runs of 138 cycles, generating high-throughput sequence data for downstream SNP discovery and genotype calling.

### SNP calling and filtering criteria

Raw marker scoring was performed using DArTsoft14, an in-house proprietary pipeline that applies sequence-based algorithms to score two types of markers: SilicoDArT markers and SNP markers. Both marker types were scored in binary format, representing the presence (1) or absence (0) of a restriction fragment carrying the marker sequence in the genomic representation of each sample. All markers were aligned to the pearl millet reference genome assembly ***PearlMillet.Tift*** [23] to enable chromosomal anchoring and positional annotation. Following alignment, a quality control filtering pipeline was applied to retain only high-confidence SNP markers for downstream analysis. Markers were filtered based on three criteria: a minor allele frequency (MAF) greater than 5%, a missing data rate per sample below 20%, and a missing data rate per SNP below 10%. SNPs failing any of these thresholds were excluded, resulting in a high-quality filtered SNP dataset used for all subsequent genomic analyses.

### Genetic diversity metrics

Genetic diversity metrics were computed for the full collection of 279 individuals using the *snpReady* package in R. For each SNP, the following statistics were estimated: minor allele frequency (MAF), expected heterozygosity (gene diversity, DG), observed heterozygosity (HO), polymorphism information content (PIC), inbreeding coefficient (F), and selection coefficient (S). Summary statistics were averaged across all retained polymorphic loci and reported by breeding source: Group 1 (international PMiGAP lines) and Group 2 (local breeding material, including check cultivars and breeding lines at various generations), as well as for the complete panel.

### Population structure analysis

Population structure was examined only within the GWAS panel made of 137 genotypes through principal component analysis and partitioning-around-medoids (PAM) [24] clustering of Euclidean distances computed from standardized SNP dosages using the *Cluster* R package. This method partitions individuals into k clusters by identifying the most representative central object, or medoid, within each cluster. The optimal number of genetic clusters (k) was determined by testing k values ranging from 2 to 8 and selecting the value yielding the highest average silhouette width. To visualize population structure, principal component analysis (PCA) was applied to the filtered genotype matrix, and a three-dimensional PCA plot was generated with PAM cluster assignments overlaid using the ***fviz_cluster ()*** function from the ***factoextra*** R package. Because structure was modelled through these continuous PCA + K terms rather than discrete cluster membership, the PAM partition is descriptive only and does not enter the GWAS model.

### Linkage disequilibrium analysis

Linkage disequilibrium (LD) decay was also assessed across the GWAS panel to characterize the extent of allelic associations among SNP markers and to inform the resolution of genome-wide association analyses. Prior to LD computation, the VCF file was converted to Genomic Data Structure (GDS) format using the snpgdsVCF2GDS function of the *SNPRelate* package [25] in R program. Pairwise LD was estimated chromosome-by-chromosome using the s*npgdsLDMat* function, which computes the Pearson correlation coefficient (r) between allele dosages at pairs of SNP loci within a sliding window of 500 consecutive markers. The squared correlation coefficient (r²) was derived and used as the primary measure of LD. Only SNP pairs separated by a physical distance of up to 500 kb were retained for downstream analysis. Pairwise r² values were subsequently binned into non-overlapping 5 kb distance classes, and the mean r² was computed within each bin to characterize the genome-wide decay trend. The physical distance at which mean r² dropped below the conventional threshold of 0.1 was used as the operational estimate of LD extent, following the recommendation of Flint-Garcia et al. (2003) [26].

### Genome-wide association analysis

Association analysis was conducted in a panel consisting of the most advanced and stable genotypes (F_5_ ISMI lines, PMiGap inbred lines and local checks). Association Analyses were performed using the *mrMLM* (Multi-locus Random-SNP-Effect Mixed Linear Model) [27] package version 4.0.2. This method integrates six multi-locus models to enhance QTL detection while controlling for false positives: (i) mrMLM, (ii) FASTmrMLM, (iii) FASTmrEMMA, (iv) pKWmEB, (v) pLARmEB, and (vi) ISIS EM-BLASSO. Population structure (PCA) and kinship (K) matrices were incorporated into all models to correct for population stratification and individual relatedness. Although mrMLM applies a default critical LOD of 3.0, we adopted a more stringent genome-wide threshold of LOD 6.5 to limit false positives. This corresponds to a Bonferroni-adjusted significance level of α ≈ 0.005 across the 14,718 markers (−log_10_(0.005/14718) ≈ 6.5). The general model used in the multi-locus mixed model framework is described in the equation below:


$$\begin{array}{c} Y=\beta_{0}+\sum \beta_{i}X_{i}+\sum \phi_{j}Z_{j}+\varepsilon \end{array}$$ (4)

Where:

- *Y* is the phenotypic trait value,
- *βo* is the intercept of the model,
- *β*_*i*_ is the effect size (regression coefficient) of the ith SNP genotype (coded as 0,1 or 2 for the number of minor alleles),
- *X*_*i*_ is the genotype score for ith SNP,
- *ϕ*_*j*_ is the effect size of the jth covariate,
- *Z*_*j*_ is the value of the jth covariate for each individual (population structure),
- *ɛ* is the error term associated with all other unmeasured factors influencing the trait.

### Candidate gene analysis

To identify putative candidate genes underlying the detected MTAs, a physical window based on LD result upstream and downstream of each significant SNP was defined using the pearl millet reference genome (Tift23D2B1-P1-P5). All annotated genes located within this window were extracted from the corresponding genome annotation file (GFF3 format) [23] and considered as candidate genes for the corresponding marker-trait association. All analyses were conducted in R version 4.5.1 using dedicated Bioconductor packages, including *GenomicRanges, rtracklayer, Biostrings, and GenomicFeatures*. Genes identified within the defined search window were retained for downstream functional annotation to further characterize their putative roles in the regulation of the traits under study.

## Results

### Analysis of variance in the diversity panel

Analysis of variance in the diversity panel revealed significant differences (p < 0.001) among genotypes for all four traits across all three sets (Table 1). Location effects were highly significant for yield, panicle length, and flowering time, but non-significant for plant height. Genotype-by-location interaction was significant for all traits (p < 0.01 to p < 0.001). Broad-sense heritability estimates ranged from 0.75 to 0.91 for grain yield and panicle length respectively.

**Table 1:** Phenotypic variation of the studied traits in the diversity panel.

	Mean Sum of Squares			
Source of variation	Yield	Plant Height	Panicle length	Flowering time
Genotype	1851103***	4481.60***	416.41***	103.93***
Location	62108884***	1305.50ns	889.90***	912.71***
GxL	585166**	756.6**	41.84***	23.48***
Residual	459090	573.70	23.83	15.52
Mean	1119	210.90	36.39	54.51
LSD (5%)	888.96	32.14	6.94	5.34
H^2^	0.75	0.86	0.91	0.82

Legend for Significance Stars: *** p ≤ 0.001; ** p ≤ 0.01; ns = non-significant

### Descriptive statistics in the early segregating material

Descriptive statistics for the four agronomic traits in the F1 and F2 generations are summarized in (Table 2). Flowering time was the least variable trait in both generations, averaging 58.7 days in the F1 (range 47–69) and 56.7 days in the F2, where it spread more widely (range 41–74). Plant height averaged 229.1 cm in the F1 (range 158–282) and 218.3 cm in the F2, with a comparable spread but a markedly wider range (133–308 cm). Panicle length followed a similar pattern, declining from 40.1 cm in the F1 (range 22–65) to 37.3 cm in the F2, where variability increased appreciably (range 20–71). Grain yield was the most variable trait: it averaged 687.0 kg ha^-1^ in the F1 and 462.1 kg ha^-1^ in the F2, but in both generations the standard deviation approached or exceeded the mean (774.0 and 443.3, respectively), and values ranged from around 100 to over 4400 kg ha^-1^. Across all the four traits, the F2 consistently spanned a wider range than the F1, consistent with the transgressive segregation expected in early-generation breeding material.

**Table 2:** Descriptive statistics for the four agronomic traits in the F1 and F2 generations.

Trait	Generation	n	Mean	SD	Range
Flowering time (days)	F1	127	58.7	4.3	47–69
	F2	296	56.7	6.2	41–74
Plant height (cm)	F1	127	229.1	26.5	158–282
	F2	296	218.3	27.4	133–308
Panicle length (cm)	F1	127	40.1	7.5	22–65
	F2	296	37.3	9.0	20–71
Grain yield (kg. Ha^-1^)	F1	127	687.0	774.0	100–4457
	F2	296	462.1	443.3	102–3348

The F1 generation was evaluated at a single site (Bambey)

The F2 generation was evaluated at four sites (Bambey, Sefa, St-Maleme, Nioro). Means, standard deviations, and ranges pool all sites.

### Phenotypic performance and variability in the GWAS panel

The frequency distributions of the four agronomic traits in the GWAS panel are presented in (Fig 1), and the corresponding descriptive statistics are summarized in (Table 3). Panicle length (Fig 1A) displayed a roughly bell-shaped distribution centered around 38–42 cm, spanning from approximately 18–63 cm, with a CV of 23.60%. Plant height (Fig 1B) showed a broad, relatively uniform distribution ranging from approximately 150–270 cm, with a concentration of genotypes in the 200–250 cm range and a CV of 12.90%. Flowering time (Fig 1C) followed an approximately normal distribution, with values ranging from around 40–72 days, the majority of genotypes flowering between 50 and 62 days, and a CV of 8.66%. Grain yield (Fig 1D) exhibited a right-skewed distribution ranging from below 500 to nearly 4000 kg/ha, with the majority of genotypes concentrated between 1000 and 2500 kg/ha and a long tail toward higher yield values, and a CV of 43.56%.

**Table 3:** Descriptive statistics and coefficient of variation (CV) for agronomic traits in the GWAS panel.

Trait	Mean	SD	CV (%)	Min	Max
Panicle Length (cm)	37.87	8.94	23.60	15.31	62.44
Plant Height (cm)	216.43	27.86	12.90	130.15	280.29
Flowering time	54.87	4.75	8.66	37.95	72.07
Grain Yield (kg/ha)	1645.25	716.67	43.56	415.20	3805.83

*SD: standard deviation; CV: coefficient of variation; Min: minimum; Max: maximum.*

**Fig 1:**
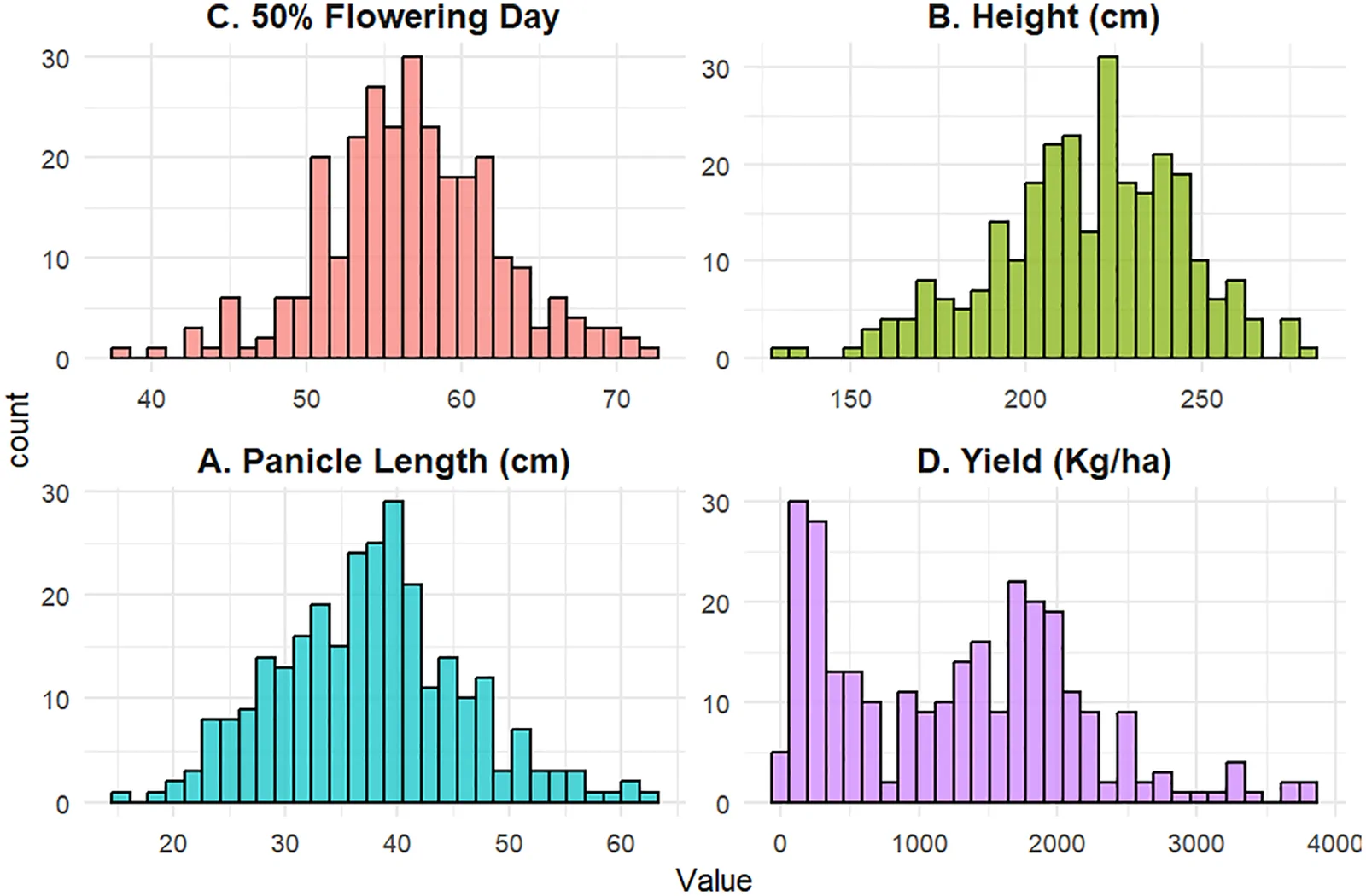
Distribution of the four studied traits across the GWAS panel.

### Trait correlations

Among the studied traits there were varying degrees of correlation (Fig 2). Plant height showed a moderate positive correlation with panicle length (r = 0.52, *p ≤ 0.001*) and grain yield (r = 0.45, *p ≤ 0.001*). Similarly, panicle length was positively correlated with yield (r = 0.28, *p ≤ 0.001*). In contrast, flowering time displayed weak correlations with other traits. It showed a slight positive correlation with plant height (r = 0.08, *p ≤ 0.001*) and panicle length (r = 0.16, *p ≤ 0.001*), but a moderate negative correlation with yield (r = –0.28, *p ≤ 0.001*).

**Fig 2:**
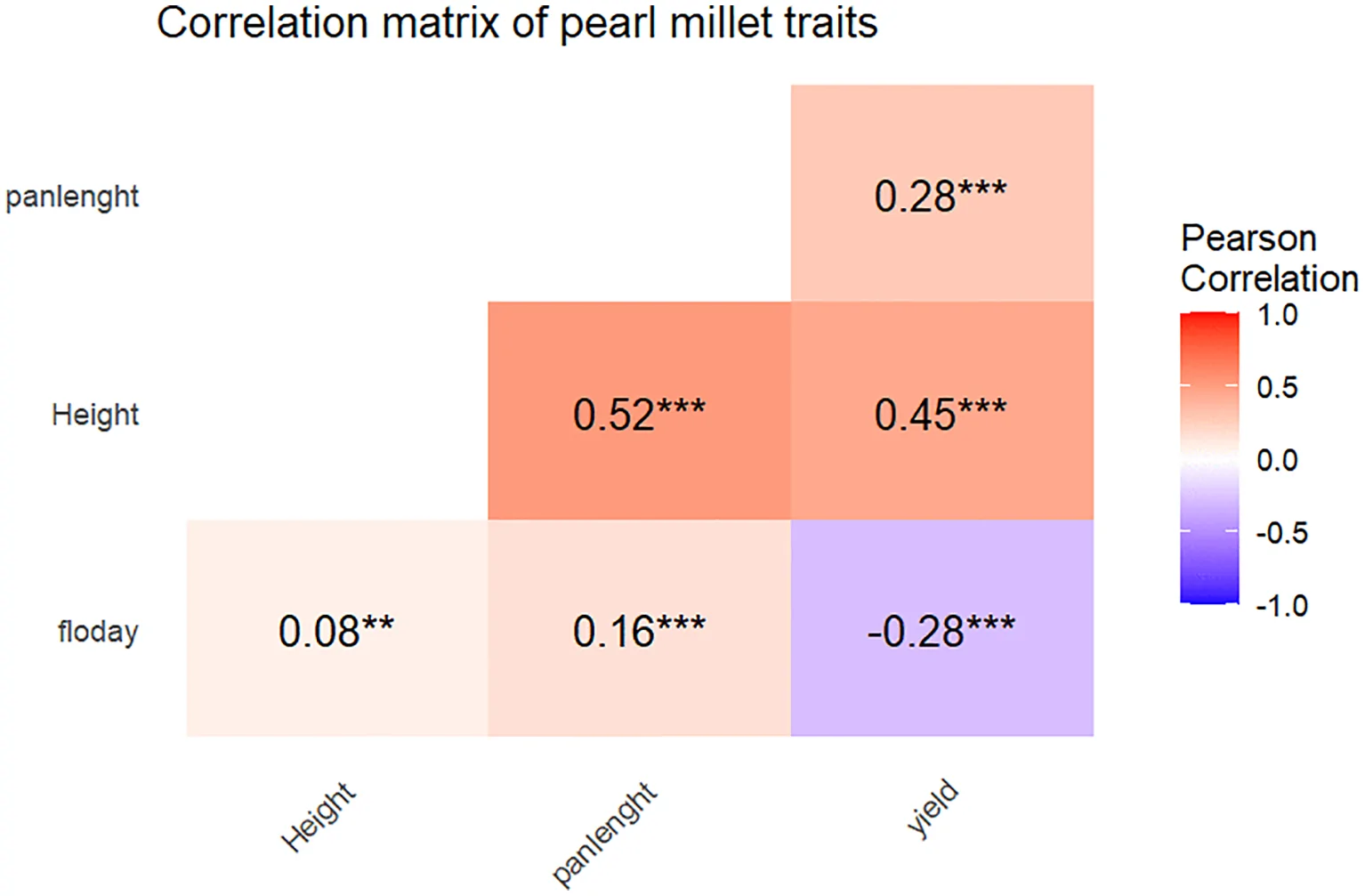
Correlation coefficients among the studied traits. Legend for Significance Stars: *** p ≤ 0.001; ** p ≤ 0.01.

### Marker coverage and distribution

The distribution of the 14718 refined SNP markers was plotted in 1 Mb (megabase pair) window across the *P. glaucum* genome (Fig 3). Variant distribution was fairly uniform across the chromosomes. The highest density was observed in the telomeric region of Chromosome 6. The total number of SNPs per chromosome ranged from 1866 SNPs on chromosome 6–2385 SNPs on chromosome 2.

**Fig 3:**
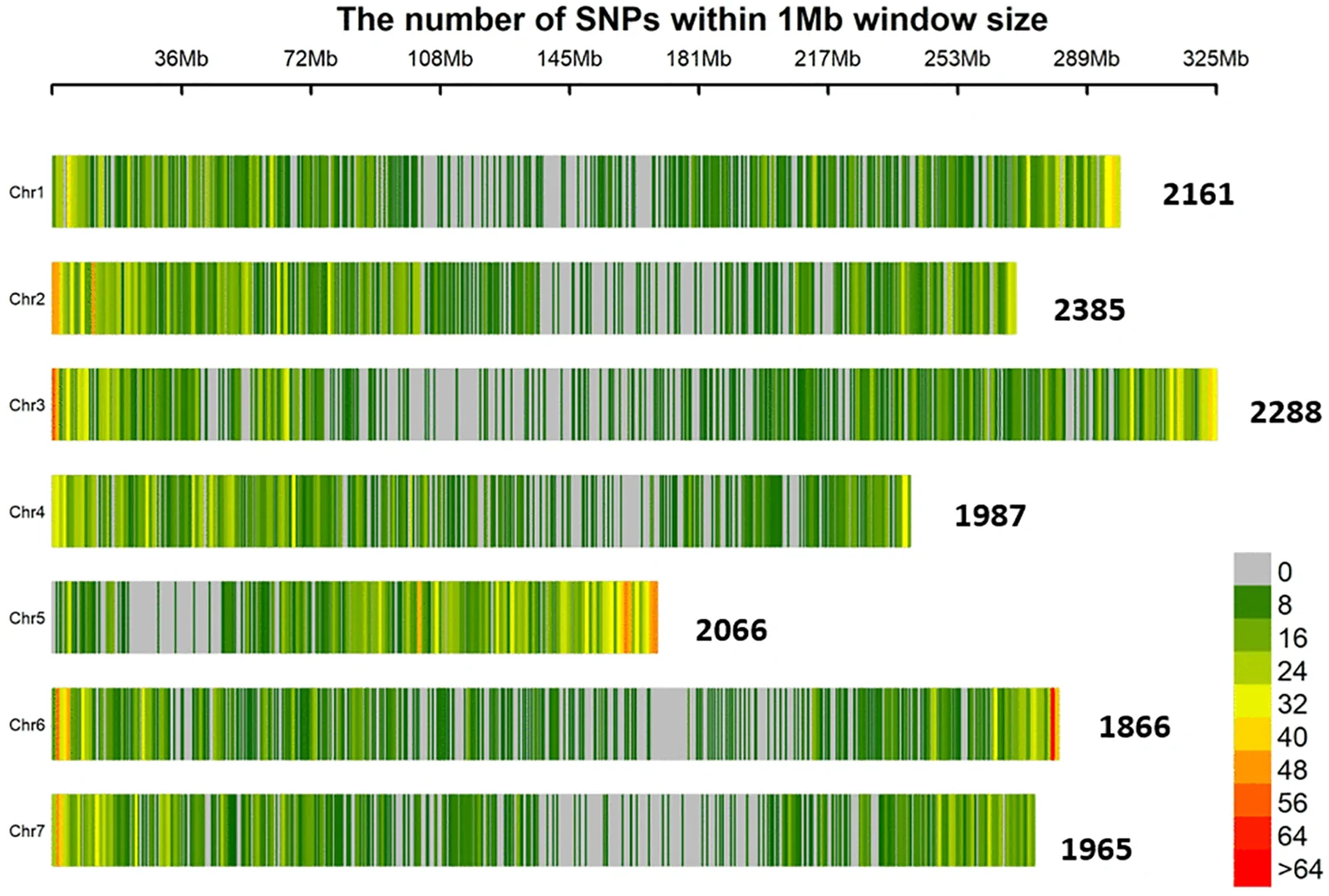
Distribution of SNPs on the 7 chromosomes of pearl millet within 1Mb window size. The horizontal axis shows chromosome length with their respective SNPs density. The legend (0- > 64) indicates the SNPs density.

### Genetic diversity metrics

Genetic diversity metrics were broadly similar across the two breeding groups and the complete collection (Table 4). Group 1 (PMiGAP) showed slightly lower values for gene diversity (DG = 0.27), PIC (0.22), and inbreeding coefficient (F = 0.29) compared to Group 2 (DG = 0.30, PIC = 0.24, F = 0.32), while MAF was nearly identical (0.20 vs 0.21). Observed heterozygosity was identical across all three groups (HO = 0.20), and the selection coefficient was slightly higher in Group 2 (S = 0.40) than in Group 1 (S = 0.38). Overall, the complete collection metrics closely mirrored those of Group 2, reflecting the larger contribution of local breeding material to the full dataset.

**Table 4:** Genetic diversity metrics estimated in the complete genotyped panel (n = 279). Group 1 (PMiGAP panel) and Group 2 (local breeding material).

Group	Diversity metric	Mean
**Group 1**	DG (gene diversity)	0.27
	PIC (Polymorphism Information Content)	0.22
	MAF (Minor Allele frequency)	0.20
	Ho (Observed heterozygosity)	0.20
	F (inbreeding coefficient)	0.29
	S (selection coefficient)	0.38
**Group 2**	**Diversity metric**	**Mean**
	DG (gene diversity)	0.30
	PIC (Polymorphism Information Content)	0.24
	MAF (Minor Allele frequency)	0.21
	Ho (Observed heterozygosity)	0.20
	F (inbreeding coefficient)	0.32
	S (selection coefficient)	0.40
**Complete panel**	**Diversity metric**	**Mean**
	DG (gene diversity)	0.30
	PIC (Polymorphism Information Content)	0.24
	MAF (Minor Allele frequency)	0.21
	Ho (Observed heterozygosity)	0.20
	F (inbreeding coefficient)	0.32
	S (selection coefficient)	0.41
		

### Population structure analysis in the complete panel

PAM clustering identified a two-group partition as the optimal solution (average silhouette width maximal at k = 2), comprising 44 and 93 entries (Fig 4). Differentiation between the two groups was nonetheless weak: the first three principal components jointly captured less than 9% of the genotypic variance, average silhouette widths remained below 0.05. The two clusters showed some overlap in principal component space. The larger group was dominated by ICRISAT-derived (PMiGAP) lines, whereas the smaller group was enriched for locally developed ISMI and Senegalese check materials, reflecting a loose tendency to group by breeding origin. Because the association model corrected for structure and relatedness through continuous PCA + kinship terms rather than cluster membership, this partition is descriptive only and had no bearing on the detected MTAs.

**Fig 4:**
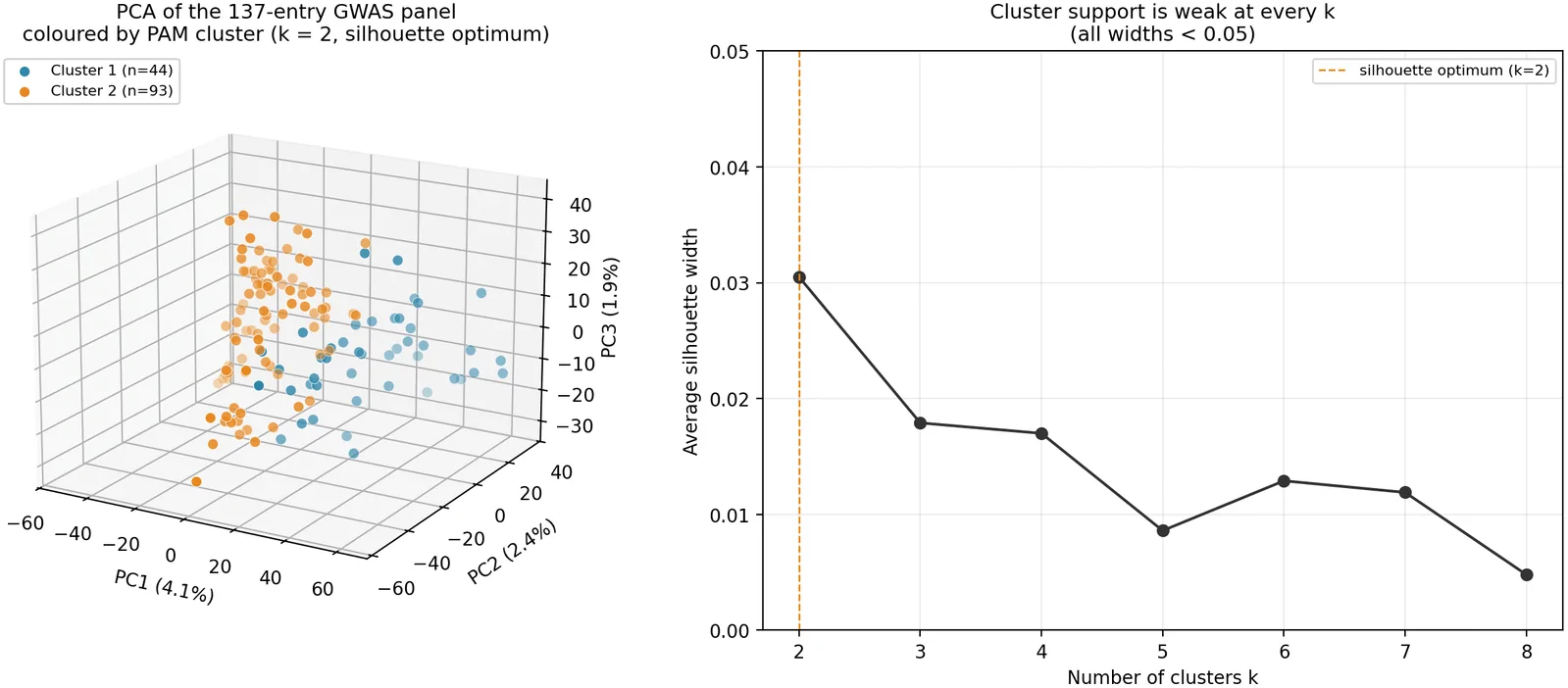
Population structure in the 137-entry GWAS panel. Left: principal component analysis of the 137 advanced entries, colored by partitioning-around-medoids (PAM) cluster at the silhouette-optimal k = 2 (Cluster 1, n = 44; Cluster 2, n = 93); the first three components explained 4.1%, 2.4%, and 1.9% of the genotypic variance and the clusters overlapped extensively. Right: average silhouette width peaked at k = 2 (dashed line) but stayed below 0.05 for all k, indicating no strongly differentiated subpopulations within the panel.

### Linkage disequilibrium analysis

Linkage disequilibrium (LD) decay was assessed across the GWAS panel by computing mean r^2^ values between all pairwise SNP combinations as a function of physical distance (kb). LD declined rapidly with increasing genomic distance, dropping from r^2^ ≈ 0.10 at near-zero distance to the conventional threshold of r^2^ = 0.1 at approximately 8 kb (Fig 5). Beyond this threshold, LD continued to decline progressively, stabilizing at a residual background level of r^2^ ≈ 0.02–0.04 beyond approximately 50 kb. The locally estimated scatterplot smoothing (LOESS) curve confirmed the overall decay trend and was consistent with the raw mean r^2^ profile.

**Fig 5:**
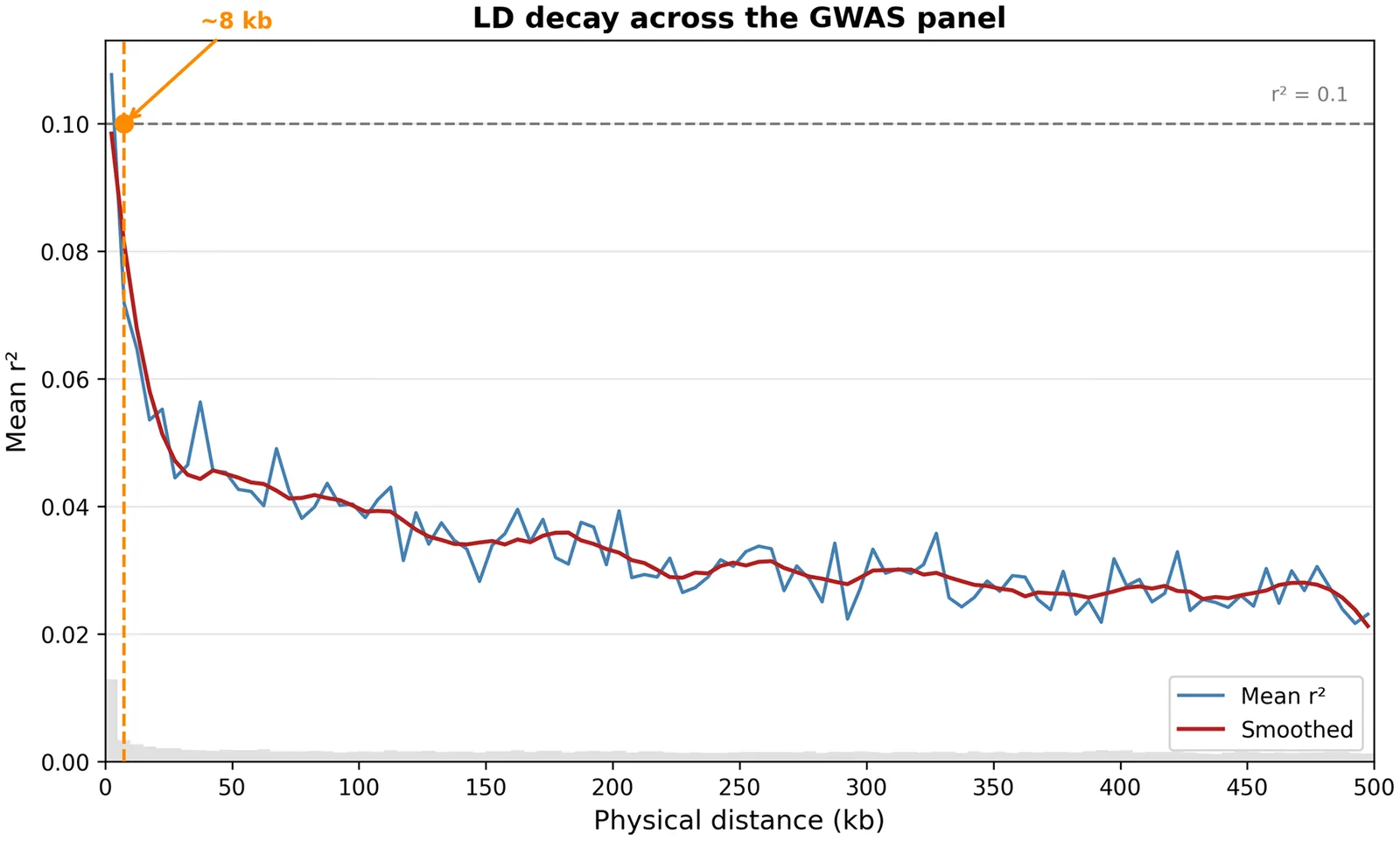
Linkage disequilibrium (LD) decay across the GWAS panel. Mean r^2^ values (blue line) are plotted against physical distance (kb) between SNP pairs. The locally estimated scatterplot smoothing (LOESS) curve (red line) highlights the overall decay trend. LD decayed below the threshold of r^2^ = 0.1 (dashed grey line) at approximately 8 kb.

### Marker effects on agro-morphological traits

Multi-locus GWAS identified a total of 11 significant marker-trait associations (MTAs) distributed across 6 of the 7 pearl millet chromosomes, with LOD scores ranging from 6.83 to 10.78 (Table 5). Grain yield was associated with three MTAs on chromosomes 2, 4, and 5, with R^2^ values ranging from 6.18% to 48.67%. Plant height showed the highest number of associated loci, with five MTAs detected on chromosomes 3, 4, 5, 6, and 7, explaining between 8.07% and 19.0% of phenotypic variance. Panicle length was associated with two MTAs on chromosomes 2 and 4, with R^2^ values of 10.52% and 13.83%. Flowering time was associated with a single MTA on chromosome 2 (SNPPM2516, LOD = 8.28, R^2^ = 5.92%). Allele effects were variable in direction across traits and loci, ranging from −660.24 to 208.25. The Manhattan and Q-Q plots for all traits are presented in (Fig 6).

**Table 5:** Marker-trait associations (MTAs) detected in chromosomal regions using multi-locus genome-wide association analysis.

Trait	Chr	SNP	Position (bp)	LOD score	Allele effect	R^2^ (%)
**Grain yield**	4	SNPPM6939	3915845	7.34	−660.24	48.67
	2	SNPPM4030	224179667	8.08	−185.23	6.18
	5	SNPPM10777	166630510	6.99	208.25	6.98
**Plant height**	6	SNPPM11926	140205667	10.78	−13.62	11.65
	7	SNPPM13293	31057406	7.86	13.67	16.34
	3	SNPPM5025	24141837	7.87	8.84	8.073
	4	SNPPM8294	128176057	7.58	−13.78	13.60
	5	SNPPM8955	47504290	6.90	−13.98	19.0
**Panicle length**	4	SNPPM8661	227900429	6.86	−3.66	13.83
	2	SNPPM3346	77584700	6.83	−4.43	10.52
**Flowering time**	2	SNPPM2516	13693642	8.28	−1.15	5.92

Chromosomal position (bp), LOD score, allele effect, and phenotypic variance explained (R^2^, %) are reported for each marker. Allele effect represents the additive effect of the alternative allele on the trait mean; positive and negative values indicate increasing and decreasing effects, respectively. R^2^ denotes the proportion of phenotypic variance explained by each SNP. LOD scores are derived from the best-fitting multi-locus model applied independently to each group.

**Fig 6:**
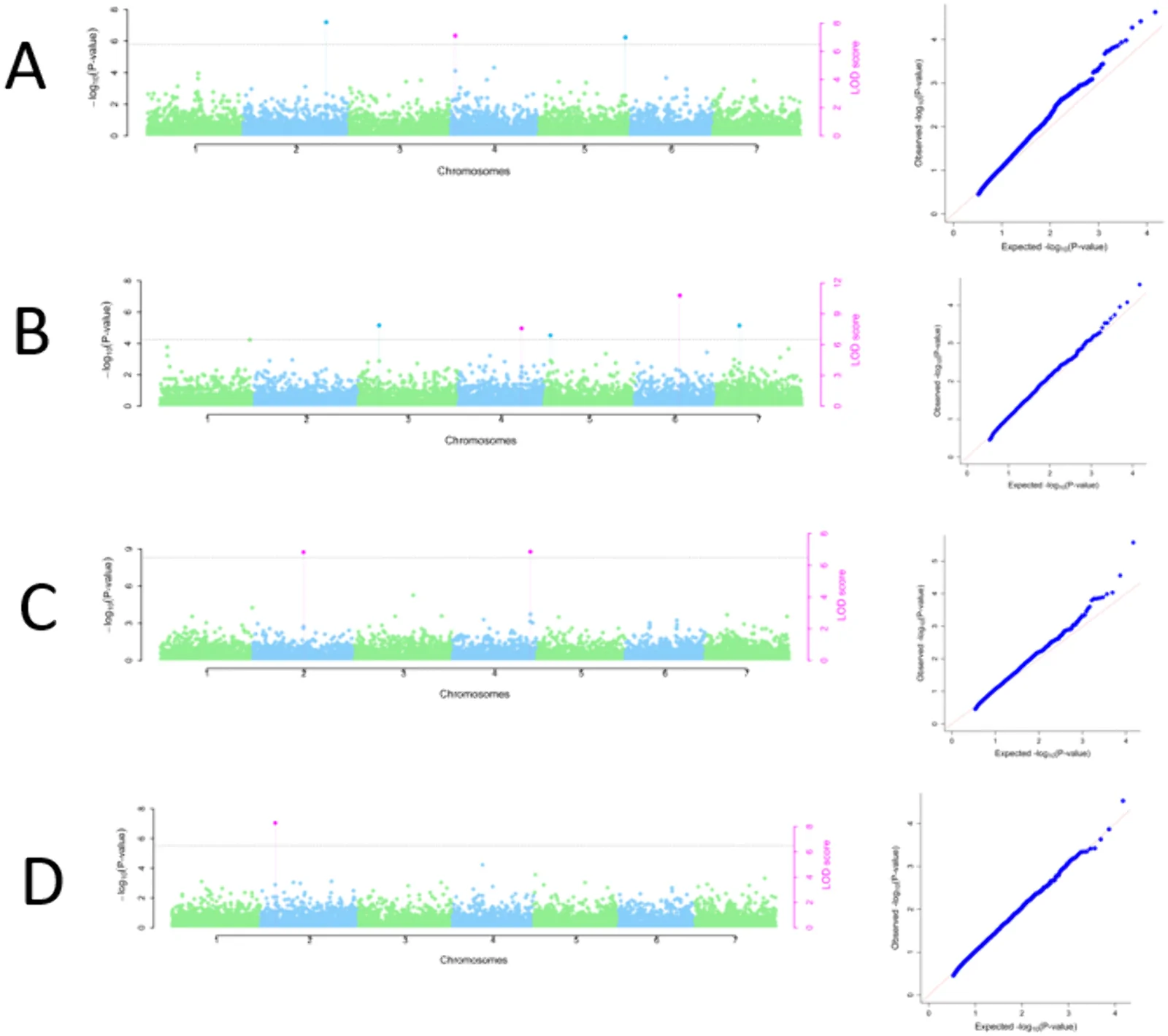
Manhattan and quantile-quantile (Q-Q) plots for the four agronomic traits. Results are shown for grain yield (A), plant height (B), panicle length (C), and flowering time (D). For each trait, the Manhattan plot (left) displays the − log_10_(p-value) for each marker across the seven chromosomes of pearl millet, with the secondary axis (pink) indicating the LOD score. The horizontal grey line represents the LOD threshold of 6.5 applied for declaring significant associations. Markers in red were identified by more than one model.

### Candidate gene analysis

To identify putative candidate genes underlying the significant MTAs, significant SNPs were annotated against the reference genome and overlapping genes were retrieved (Table 6); functional annotations were inferred by homology and, where indicated, transferred from related species such as *Setaria italica*, and should therefore be read as positional hypotheses rather than demonstrated causal genes. For grain yield, associations on chromosome 4 co-localized with two receptor-like kinase genes, a cysteine-rich receptor-like protein kinase 6 (dpca4g263180) and a DUF26/TKL_IRAK kinase (dpca4g263190), whose families have been implicated in stress perception and signal transduction, while on chromosome 5 significant markers overlapped an expansin-A12 gene (dpca5g385240), annotated as a cell-wall-loosening protein linked to cell expansion and grain development, and a fatty acid–CoA ligase (dpca5g385250) involved in lipid metabolism.

**Table 6:** Candidate genes directly overlapping significant SNPs identified in the pearl millet panel. Gene positions are based on the reference genome annotation, and only SNPs located within 8 kb window annotated gene regions are presented.

Trait	Chr	Gene width	Gene ID	Function
**Grain Yield**	Chr04	2371	dpca4g263180.840	PREDICTED: *Setaria italica* cysteine-rich receptor-like protein kinase 6 (LOC101765034), mRNA
	Chr04	3071	dpca4g263190.840	protein TKL_IRAK_DUF26-ld.3 - DUF26 kinases have homology to DUF26 containing loci; expressed(gfam000122) | Protein containing Pkinase; Pkinase_Tyr; Stress-antifung domains
	Chr05	2184	dpca5g385240.840	PREDICTED: expansin-A12 (gfam000082) | Protein containing DPBB_1; Pollen_allerg_1 domains
	Chr05	3600	dpca5g385250.840	Fatty acid CoA ligase (gfam000054) | Protein containing AMP-binding; AMP-binding_C domains
**Plant height**	Chr06	3086	dpca6g429350.840	hydroxyproline-rich glycoprotein family protein(gfam007992)
	Chr06	1574	dpca6g429360.840	PREDICTED: LOW-QUALITY PROTEIN: protein RKD5-like (gfam001579) | Protein containing RWP-RK domains
	Chr07	2538	dpca7g479100.840	Isocitrate lyase (gfam003517) | Protein containing ICL domains
	Chr04	698	dpca4g304390.840	Function unknown XXXXX (gfam010611)
**Panicle length**	Chr04	3509	dpca4g323040.840	Pathogenesis-related transcriptional factor/ERF; DNA-binding protein XnoCX(gfam011115) | Protein containing AP2 domains
	Chr04	252	dpca4g323050.840	Integrase core domain protein
	Chr02	2103	dpca2g122560.840	progastricsin (pepsinogen C) (gfam000023) | Protein containing Asp; TAXi_C; TAXi_N domains
**Flowering time**	Chr02	1563	dpca2g096240.840	Indole-3-acetate beta-glucosyltransferase XnoC3(gfam009042)

For plant height, candidate genes included a hydroxyproline-rich glycoprotein (dpca6g429350) and an RWP-RK/RKD5-like transcription factor (dpca6g429360) on chromosome 6, annotated with roles in cell-wall structure and developmental regulation, together with an isocitrate lyase (dpca7g479100) on chromosome 7 and an additional chromosome 4 gene of unknown function (dpca4g304390).

For panicle length, a notable candidate was an AP2/ERF transcription factor on chromosome 4 (dpca4g323040), a family with established roles in inflorescence and organ development, whereas a second chromosome 4 locus corresponded to a transposable-element-derived integrase domain (dpca4g323050) and a chromosome 2 marker overlapped an aspartic-protease/xylanase-inhibitor gene (dpca2g122560).

For flowering time, a single significant association on chromosome 2 co-localized with an indole-3-acetate β-glucosyltransferase (dpca2g096240), a gene annotated in auxin homeostasis during developmental transitions. Chromosome 4 thus carried candidate genes for grain yield, plant height, and panicle length; as noted above, however, these lie at widely separated positions and represent physically independent loci rather than a single pleiotropic region. Among the annotated candidates, the developmental genes expansin-A12, the AP2/ERF transcription factor, and the auxin-conjugating β-glucosyltransferase offer the most plausible functional hypotheses for the associated agronomic traits and are accordingly prioritized for validation.

## Discussion

### Phenotypic performance analysis

Analysis of variance revealed highly significant genotypic effects for all four studied traits in the diversity panel, confirming the presence of exploitable genetic variation in the evaluated pearl millet panel. Similar significant genotypic differences for these traits have been reported in previous pearl millet studies conducted across West African environments [9,28]. The significant genotype × location (G × L) interaction detected for all traits underlines the sensitivity of these traits to environmental variation, a finding consistent with reported multi-environment analyses of pearl millet and sorghum agromorphological performances [29].

The levels of phenotypic variability observed in this study are consistent with those reported in pearl millet diversity panels evaluated across West Africa and the broader semi-arid tropics [12,30], confirming that the Senegalese germplasm captures a representative range of genetic diversity for the traits studied. The high CV for grain yield reflects the phenotypic heterogeneity of the panel entries rather than residual segregation; this heterogeneity is expected given that the panel draws on entries from different breeding sources. The relatively low variation in flowering time compared to yield-related traits follows a pattern widely documented in pearl millet, where photoperiod sensitivity and adaptation to terminal drought impose strong directional selection on phenology while leaving morphological and yield traits more open to diversification [31].

Heritability estimates across traits were consistent with the quantitative genetic architecture expected for pearl millet under Sahelian conditions. The relatively high *H*^2^ for grain yield indicates a substantial genetic basis for yield variation in this panel, comparable to values reported from combining ability and multi-environment studies in West African pearl millet [32], though the residual environmental component underscores the importance of multi-environment evaluation for reliable selection gains. The high heritability observed for panicle length and plant height reflects their predominantly additive genetic architecture and lower sensitivity to G × E interaction, consistent with findings across recombinant inbred line populations and diversity panels in pearl millet [33,34]. The moderate to high heritability for flowering time aligns with reports from Senegalese landraces [35], where photoperiod sensitivity introduces trial-to-trial variation while maintaining a meaningful genetic signal. These estimates suggest that direct phenotypic selection will be most efficient for morphological traits, while grain yield improvement will require multi-environment testing and genomic-assisted approaches to effectively capture and exploit the available genetic variation.

### Phenotypic distribution and trait correlation

The phenotypic distribution across the GWAS panel revealed substantial variation for all four traits, consistent with quantitative inheritance and phenotypic ranges reported in pearl millet panels evaluated across West Africa and the semi-arid tropics [12,30]. The correlation structure reflected biologically meaningful associations consistent with patterns widely documented in pearl millet: the positive relationships among plant height, panicle length, and grain yield suggest shared developmental pathways and pleiotropic genetic control, while the negative correlation between flowering time and grain yield reflects the drought escape mechanism whereby early flowering confers yield stability under terminal drought, a priority selection criterion in Sahelian breeding programs [31]. These patterns support the use of indirect selection through morphological traits under favorable conditions and confirm early flowering as a key target for adaptation to the Senegalese rainfall pattern.

### Genetic diversity metrics, population structure

The genetic-diversity indices and the population-structure analysis were computed on complementary subsets rather than on a single panel, and are best read together. Gene diversity, PIC, observed heterozygosity, and inbreeding coefficients were estimated on the full genotyped set (279 genotypes), which still included early-generation segregating material, whereas the PAM/PCA structure analysis was restricted to the 137 advanced, genetically stable entries that formed the association panel. The moderate gene diversity (GD) and PIC observed in both groups are consistent with previous SNP-based characterizations of pearl millet germplasm, where these indices typically range from 0.20 to 0.40 depending on origin, breeding history, and marker density [12,36]. The slightly lower diversity in Group 1 (PMiGAP inbreds) relative to Group 2 reflects the cumulative effect of directional selection within the ICRISAT breeding program, since elite pools under sustained improvement predictably harbour narrower allelic ranges than locally adapted or multi-cross material [37]. Group 2, by contrast, is compositionally heterogeneous: check cultivars alongside multi-cross lines spanning early to advanced generations, and this breadth sustains higher allelic diversity through the accumulated recombination and residual segregation characteristic of early-generation material, accounting for its elevated GD and PIC. These contrasts bear directly on how heterozygosity was interpreted and how the association panel was assembled. The near-identical observed heterozygosity across the two groups is consistent with the allogamous, protogynous reproductive biology of pearl millet, and the moderate inbreeding coefficients confirm that the genotyped diversity set had not reached fixation. Because non-fixed genotypes violate the fixed-line assumption underlying single-marker association testing and can inflate association noise, the early-generation segregating material was excluded and the GWAS was restricted to the 137 stable entries; residual relatedness within that panel was then controlled through a kinship matrix in the mixed-model framework [38,39]. Finally, although the PIC values fall at the lower end of the moderately informative range [40], they remain adequate for association analysis given the large number of markers retained after quality filtering, while the minor-allele-frequency spectrum spans rare variants of potential relevance to adaptation in the Sahelian environment.

Population structure within the 137-entry association panel was weak and largely continuous. PAM clustering identified *k* = 2 as the silhouette optimum, yet average silhouette widths remained below 0.05 across all values of *k*, indicating that the panel does not resolve into sharply differentiated subpopulations. This near-continuous stratification is consistent with the panel’s composition, in which PMiGAP lines and local multi-cross material derived from ICRISAT parents share substantial ancestry rather than forming discrete pools. Soft genetic boundaries of this kind are expected in an obligate outcrosser, where protogynous flowering and wind pollination sustain continuous gene flow that prevents sharp discontinuities [41,42]. The low variance captured by individual principal components is likewise a known property of high-dimensional differentiation in outcrossing cereals rather than a shortcoming of the panel, and has been reported for comparable West African germplasm [36,37]

Methodologically, continuous stratification of this form favours fitting several leading PCs as fixed covariates together with a kinship matrix as a random effect, rather than a single Q-matrix, to control false positives while preserving detection power [43]. The panel therefore sits at a productive point in the diversity–stratification trade-off: its allelic range is broad enough to power association detection, while its structure is correctable rather than confounding. Association signals recovered consistently across the panel are expected to be the most transferable across the ISRA–ICRISAT breeding continuum. Finally, the faster LD decay characteristic of West African pearl millet relative to South Asian material [12,36] means associations from Indian panels do not transfer directly to this context, reinforcing the value of a regionally anchored reference panel for future multi-trait and multi-environment GWAS in the Sahelian production system.

### Linkage disequilibrium analysis

Linkage disequilibrium decayed rapidly, with mean r^2^ falling below 0.1 at approximately 8 kb across the 14,718 SNPs scored in the GWAS panel, consistent with the outcrossing biology of pearl millet, in which recurrent recombination erodes marker–marker correlations over short distances. The reference PMiGAP diversity panel reported even faster decay (r^2^ < 0.2 within 84–444 bp), a rate comparable to allogamous maize [11]. The greater LD extent observed here is expected from the panel’s composition: unlike a largely panmictic diversity set, our panel combined advanced inbred lines with locally derived breeding material, several entries sharing pedigree and subject to directional selection, both of which reduce effective recombination and extend haplotype blocks. Such subpopulation-dependent variation is documented in pearl millet, where the West-African subgroup shows greater decay owing to a longer recombination history [36]. The short-range LD confirms that dense, genome-wide marker coverage is required to capture causal variants [44].

### Marker effects on agro-morphological traits and candidate gene analysis

Eleven MTAs were detected across six chromosomes for the traits under study, each association individually explaining a modest fraction of the overall phenotypic variance. This distribution is characteristic of the polygenic, small-effect architecture typical of quantitative agronomic traits in pearl millet, and it aligns with prior genome-wide work in the crop: the reference GWAS across test-cross progenies identified 1,054 significant MTAs for fifteen traits, with individual associations explaining between 9% and 27% of phenotypic variation [11]. The only exception in our study was on chromosome 4, where the marker SNPPM6939 associated with grain yield explained 48.67% of the phenotypic variance and had a negative effect on yield. An effect of this magnitude is atypical for a quantitative trait and should be interpreted with caution: in a panel of only 137 entries with no independent validation population, single-locus effect sizes are prone to substantial overestimation (the Beavis effect), and the value may partly reflect minor-allele frequency or allele-class imbalance rather than a true major effect. This need for independent confirmation is reinforced by the deleterious-variant burden documented in Senegalese pearl millet [45], which argues for building association resources on regionally adapted germplasm and validating candidate signals before they are deployed in selection.

Several of other associations correspond, at the chromosome level, to genomic regions previously implicated in the same traits. Earlier marker-based studies in pearl millet associated marker alleles with plant height on linkage group 7, and with panicle length on linkage groups 2, 5, and 7, while a further allele on linkage group 7 was associated with grain yield [44], and more recent association analyses have likewise recovered agronomic-trait associations for plant height, panicle length, and grain weight in the crop [46]. This convergence of independent panels and marker systems on broadly overlapping regions is consistent with these being genuine effects rather than artefacts; the correspondence is, however, at the chromosome level and does not by itself establish that the same underlying loci are involved.

A notable feature of the present results is that chromosome 4 harbored associations for three traits: grain yield (SNPPM6939, 3.9 Mb), plant height (SNPPM8294, 128.2 Mb), and panicle length (SNPPM8661, 227.9 Mb), making it, together with chromosome 2, the most trait-rich region in the panel. This co-occurrence does not, however, imply a shared genetic basis. The three markers are dispersed across more than 220 Mb of the chromosome, with the plant-height and panicle-length signals alone separated by roughly 100 Mb. Because linkage disequilibrium in this panel decayed to r^2^ < 0.1 within approximately 8 kb, associations this far apart cannot be in linkage with one another and instead represent physically independent loci that merely reside on the same chromosome. The co-localization of plant height and panicle-related traits reported previously in pearl millet [47] therefore does not extend to the present associations, which show no evidence of a single pleiotropic region governing multiple traits. The functional candidates underlying each of these independent signals are considered below. Inspection of the ± 8 kb intervals flanking the significant markers nominated several functionally credible candidates, which should be regarded as hypotheses for subsequent validation rather than confirmed causal genes. Because these candidates were identified solely by physical proximity to significant markers within LD-defined windows, their positions localize but do not establish a causal relationship, and the links proposed below are positional hypotheses to be tested functionally. Two gene families recurred in the intervals flanking the plant-architecture and panicle associations: F-box domain proteins and AP2/ERF transcription factors. The AP2/ERF family is a well-characterized regulator of inflorescence development and yield in cereals: in rice, these transcription factors participate in the regulation of panicle architecture, grain plumpness, and yield formation by controlling inflorescence meristem activity, cell division, and differentiation [48], and their agronomic relevance extends across grasses, in wheat, mutation of an AP2/ERF gene (DUO-B1) regulating spike architecture increased grain number per spike and raised field yield without penalizing other major agronomic traits [49]. The presence of an AP2/ERF candidate near the chromosome 4 region jointly associated with plant height and panicle length would therefore be consistent with a role in meristem determinacy and inflorescence patterning, though this remains to be demonstrated. The F-box candidates are similarly noteworthy: in rice, mutations in the F-box gene LARGER PANICLE improve panicle architecture and enhance grain yield [50], and non-synonymous variants in both an AP2/ERF gene and an F-box domain-containing protein were recently reported to jointly explain a large proportion of the variance for panicle exsertion [51]. The recurrence of these two families in our intervals parallels that functional pairing and supports their prioritization as candidates for follow-up, rather than confirming their involvement in the panicle-related associations.

For flowering time, the strongest a priori candidate in pearl millet is the phytochrome pathway. Natural variation at PHYTOCHROME C is a long-recognized determinant of phenology and morphology in the crop: association studies first linked natural variation at PHYC to flowering time and morphological variation across a set of inbred lines [41], a result subsequently refined through fine-scale association mapping and analysis of linkage disequilibrium and selection in the vicinity of the PHYTOCHROME C gene [52]. This locus has since been repeatedly implicated in Sahelian adaptation, having been identified as a major flowering-time gene underpinning selection for earlier flowering in response to climatic variation [31]; it is cited here as regional context, as the flowering association recovered in the present panel does not fall within the PHYC region. Flowering time is highly quantitative [53], so the single association recovered here most likely reflects the limited power of a panel of this size under a stringent multi-locus threshold, rather than a truly simple genetic basis, with a larger panel expected to resolve more loci. It is nonetheless tempting to speculate that crosstalk between photoreceptor and auxin-mediated pathways contributes to the phenological variation observed here, a hypothesis consistent with documented phytochrome–auxin interactions in other species [54] but one that would require direct functional testing to validate.

## Conclusion

This study reported a genome-wide association analysis of grain yield, plant height, panicle length, and flowering time in a Senegalese pearl millet panel combining internationally derived inbred lines with locally adapted breeding material. Eleven MTAs were detected across six chromosomes, several linked to genes plausibly involved in cell expansion, inflorescence development, and hormone-mediated growth. Because associations from South Asian panels transfer poorly to West African germplasm, these results are of particular regional value, with the plant height and panicle length loci and the large-effect grain-yield locus on chromosome 4 emerging as the most robust and promising targets for marker-assisted and genomic selection. Realizing that potential will depend on functional validation of the candidate genes and confirmation of the associations in larger, stabilized panels spanning a wider range of West African environments. This case is reinforced by the burden of deleterious variants carried in Senegalese pearl millet germplasm, which argues both for confirming candidate signals on this genetic background and for using maturing genomic resources to track and progressively purge these alleles from the breeding pool while preserving its regional adaptation. Carried forward into multi-environment testing, genomic-selection pipelines, and regionally important constraints such as downy mildew and Striga, these findings offer a grounded basis for genomics-assisted pearl millet improvement in Senegal and the broader Sahel.

## Supporting information

S1 Table All genotypic data. (TXT)

S2 Table All phenotypic data. (TXT)

S3 Table GWAS panel cluster assignments. (CSV)

S4 Table Phenotypic data GWAS panel. (CSV)

S5 Table Genotypic data GWAS panel. (CSV)

S6 Table Plant material info. (XLSX)
